# Advancing Pediatric Sports Medicine: A Review of the 4th Edition of the Oxford Textbook of Children’s Sport and Exercise Medicine, Edited by Neil Armstrong and Willem Van Mechelen and Published by Oxford University Press

**DOI:** 10.1186/s40798-024-00748-y

**Published:** 2024-10-14

**Authors:** Robert Sallis

**Affiliations:** Clinical Professor of Family Medicine, Kaiser Permanente Bernard J. Tyson School of Medicine, Kaiser Permanente Medical Center, 9985, Sierra Ave, Fontana, CA 92335 USA

**Advancing Pediatric Sports Medicine: A Review of the 4th Edition of the Oxford Textbook of Children’s Sport and Exercise Medicine**,** edited by Neil Armstrong and Willem van Mechelen and published by Oxford University Press. (ISBN 978-0-19-284396-8)** [1]

As a sports medicine physician involved in the care of young athletes, I am honored to review the fourth edition of the Oxford Textbook of Children’s Sport and Exercise Medicine. With each successive edition, this landmark textbook has continued to set the standard for excellence in pediatric sports medicine, and the newest edition does not disappoint. In this review, I’ll delve into the myriad reasons why this edition is a must-have resource for medical practitioners, scientists, allied health professionals, coaches, educators, and students interested in youth sport and pediatric exercise science and medicine.

## Up-to-Date Comprehensive References with a Sound Scientific Foundation:

At its core, the latest edition of the Oxford Textbook of Children’s Sport and Exercise Medicine remains a comprehensive reference text rooted in a sound scientific evidence-based foundation. It serves as a guiding light, supporting and challenging readers to deepen their understanding of pediatric sports medicine. With each chapter comprehensively referenced, readers are empowered to explore topics in depth, fostering critical inquiry and scholarly engagement. With sections on Exercise Science, Exercise Medicine, Sports Science, and Sports Medicine, this textbook takes the reader from “bench to bedside/sideline” through the application of the latest basic science to clinical practice.

## Critical Analysis and Identification of Knowledge Gaps:

Building on the strengths of previous editions, this edition continues to critically analyze the research literature, identifying what we know, gaps in our knowledge, and challenging prevailing dogma. By encouraging readers to question assumptions and explore new avenues of inquiry, this textbook will help drive advancement in the field.

## Integration of Experimental Techniques and Technologies:

A notable feature of this latest edition is its exploration of recently developed experimental techniques, technologies, and methods of interpreting data. These advancements have provided new insights into the complex relationships between physical activity, exercise, physical fitness, health, and sport performance in childhood and adolescence. By incorporating the latest innovations, the textbook ensures readers are equipped with cutting-edge knowledge to address contemporary challenges in pediatric sports medicine.

## Extensive Referencing and Cross-Referencing:

True to its tradition, all chapters in this edition have been updated and are extensively referenced, promoting further study and scholarly exploration. Moreover, the textbook is cross-referenced within and across sections, facilitating easy access to complementary information. This interconnectedness enhances the reader’s ability to integrate diverse perspectives and synthesize complex concepts, fostering a holistic understanding of pediatric sports medicine.

## Global Expertise and Interdisciplinary Collaboration:

With contributions from 104 internationally recognized experts representing 67 universities and university hospitals in 25 countries, this edition embodies the global nature of pediatric sports medicine. The contributing authors represent an international “Who’s Who” in the field of Children’s Sport and Exercise Medicine and their diverse perspectives and experiences enrich the content and reflect the multidisciplinary nature of the field. Through interdisciplinary collaboration, the textbook fosters a comprehensive and nuanced approach to caring for young athletes.

## Continual Expansion and Evolution:

The latest edition of the Oxford Textbook of Children’s Sport and Exercise Medicine denotes the significant expansion and evolution of the field. With 45 new contributors and chapters on 20 new topics, the textbook reflects the dynamic nature of pediatric sports medicine and the growing interest in emerging areas of research and practice. Previous chapters have been re-appraised and re-written, often by new contributors who have emerged as leaders in their respective fields, ensuring that the content remains relevant and cutting edge. This commitment to continual evolution and expansion in this new edition reflects the dynamic nature of pediatric sports medicine and the ever-expanding body of knowledge in the field.

## Legacy of Excellence and Recognition:

Since its inception in 1999, the Oxford Textbook of Children’s Sport and Exercise Medicine has been synonymous with excellence in pediatric sports medicine. Its legacy of excellence is underscored by its recognition as the definitive text in the field and its “Highly Commended” status and short-listing for the BMA Medical Book of the Year. With each edition, the textbook continues to raise the bar for scholarship and innovation in pediatric sports medicine.

## Anticipating Future Trends and Needs:

The significant increase in contributors, universities, and countries participating in this textbook over the four editions reflects the prescient vision of the editors in predicting the growth of interest in children’s sports and exercise medicine. Furthermore, the alignment of the textbook with IOC Consensus Statements underscores its relevance and influence within the broader sports medicine community. As pediatric sports medicine continues to evolve, the Oxford Textbook of Children’s Sport and Exercise Medicine remains at the forefront, anticipating future trends and needs in the field.

## In Conclusion:

This newest edition of the Oxford Textbook of Children’s Sport and Exercise Medicine reaffirms its status as the definitive resource in pediatric sports medicine care. With its up-to-date content, sound scientific foundation, and global perspective, it is an indispensable resource for anyone involved in the care of young athletes. Whether you’re a seasoned practitioner, a budding researcher, or a student eager to explore the field, this textbook is an essential companion on the journey to excellence in pediatric sports medicine.

## Data Availability

Not applicable.
